# Supplementary material for: Costs and cost-effectiveness of community health worker programs focussed on HIV, TB and malaria infectious diseases in low- and middle-income countries (2015–2024): A scoping literature review
Source: PLOS Glob Public Health. 2025 May 9;5(5):e0004596. doi: 10.1371/journal.pgph.0004596 (PMC12063845; doi:10.1371/journal.pgph.0004596)
Supplement: S1 Table — (DOCX) [file pgph.0004596.s003.docx]

**S1 Table**

**Full Search Strategies**

|  | **Ovid MEDLINE(R) and Epub Ahead of Print, In-Process, In-Data-Review & Other Non-Indexed Citations and Daily (1946 to July 06, 2023)** | |
| --- | --- | --- |
| 1 | volunteers/ | 10847 |
| 2 | Hospital Volunteers/ | 1323 |
| 3 | health auxiliar*.tw,kw. | 46 |
| 4 | peer group*.tw,kw. | 3470 |
| 5 | health visitor*.tw,kw. | 2779 |
| 6 | Doulas/ | 224 |
| 7 | doula*.tw,kw. | 535 |
| 8 | douladural*.tw,kw. | 1 |
| 9 | ((lay or voluntary or volunteer* or untrained or unlicensed or non-professional* or nonprofessional* or informal or non-formal) adj5 (worker* or visitor* or attendant* or aide* or support* or person* or helper* or carer* or caregiver* or care giver* or consultant* or assistant* or staff or visit* or midwife or midwives or provider* or practitioner*)).tw,kw. | 22264 |
| 10 | Allied Health Personnel/ | 13030 |
| 11 | Community Health Workers/ | 6603 |
| 12 | paramedics/ | 66 |
| 13 | (paraprofessional* or paramedic* or allied health personnel or allied health worker* or support worker* or home health aide*).tw,kw. | 13109 |
| 14 | (trained adj3 (volunteer* or health worker* or mother*)).tw,kw. | 2394 |
| 15 | ((communit* or village* or frontline*) adj3 (health worker* or health care worker* or healthcare worker* or distributor* or worker* or provider*)).tw,kw. | 16975 |
| 16 | (communit* adj3 (volunteer* or aide* or support)).tw,kw. | 12108 |
| 17 | ((birth or childbirth or labor or labour) adj1 (attendant* or assistant*)).tw,kw. | 2710 |
| 18 | montrice*.tw,kw. | 0 |
| 19 | ((lay or peer) adj1 (volunteer* or mentor* or counsel* or support or intervention*)).tw,kw. | 9306 |
| 20 | (church based adj3 (intervention* or program* or counsel*)).tw,kw. | 196 |
| 21 | (linkworker* or link worker*).tw,kw. | 149 |
| 22 | barefoot doctor*.tw,kw. | 147 |
| 23 | ((health or healthcare) adj1 outreach).tw,kw. | 535 |
| 24 | Home Care Services/ | 36341 |
| 25 | (home adj1 (care or aide* or nursing or support or intervention* or treatment* or visit*)).tw,kw. | 60093 |
| 26 | ((care or aide* or nursing or support or intervention* or treatment* or visit*) adj3 (lay or volunteer* or voluntary)).tw,kw. | 5985 |
| 27 | (Auxiliary adj3 (worker* or nurse* or midwives or midwife)).tw,kw. | 728 |
| 28 | (expert patient* or health promoter* or health extension worker* or mentor mother*).tw,kw. | 1619 |
| 29 | or/1-28 | 185783 |
| 30 | Economics/ | 27505 |
| 31 | exp "Costs and Cost Analysis"/ | 265168 |
| 32 | Economics, Nursing/ | 4013 |
| 33 | Economics, Medical/ | 9249 |
| 34 | exp "Fees and Charges"/ | 31382 |
| 35 | exp Budgets/ | 14119 |
| 36 | budget*.ti,ab,kf. | 36039 |
| 37 | (economic* or cost or costs or costly or costing or price or prices or pricing or expenditure or expenditures or expense or expenses or financial or finance or finances or financed).ti,kf. | 279577 |
| 38 | (economic* or cost or costs or costly or costing or price or prices or pricing or expenditure or expenditures or expense or expenses or financial or finance or finances or financed).ab. /freq=2 | 380289 |
| 39 | (cost* adj2 (effective* or utilit* or benefit* or minimi* or analy* or outcome or outcomes)).ab,kf. | 209257 |
| 40 | (value adj2 (money or monetary)).ti,ab,kf. | 3037 |
| 41 | exp models, economic/ | 16224 |
| 42 | economic model*.ab,kf. | 4209 |
| 43 | exp Decision Theory/ | 13301 |
| 44 | (decision* adj2 (tree* or analy* or model*)).ti,ab,kf. | 37770 |
| 45 | Disability-Adjusted Life Years/ | 188 |
| 46 | (Disability-Adjusted Life Years or DALYS).tw,kw. | 4950 |
| 47 | quality-adjusted life years/ | 15725 |
| 48 | (quality-adjusted life years or QALYS).tw,kw. | 13150 |
| 49 | Return on investment.tw,kw. | 2452 |
| 50 | (multiple criteria decision analysis or MCDA).tw,kw. | 972 |
| 51 | Cost-Benefit Analysis/ | 92689 |
| 52 | cost-benefit analysis.tw,kw. | 5619 |
| 53 | (Economic evaluation* or economic analys*).tw,kw. | 23537 |
| 54 | Financial management/ | 17074 |
| 55 | (Financial planning or financial management).tw,kw. | 1692 |
| 56 | or/30-55 | 824735 |
| 58 | 29 and 56 and 57 | 3870 |
| 59 | limit 58 to yr="2015 -Current" | 1832 |
| **Embase Classic+Embase (1947 to 2023 July 07)** | | |
| 1 | volunteer/ | 66214 |
| 2 | Hospital Volunteer/ | 165 |
| 3 | health auxiliar*.tw,kw. | 71 |
| 4 | peer group*.tw,kw. | 4548 |
| 5 | health visitor*.tw,kw. | 3182 |
| 6 | Doula/ | 367 |
| 7 | doula*.tw,kw. | 544 |
| 8 | douladural*.tw,kw. | 1 |
| 9 | ((lay or voluntary or volunteer* or untrained or unlicensed or non-professional* or nonprofessional* or informal or non-formal) adj5 (worker* or visitor* or attendant* or aide* or support* or person* or helper* or carer* or caregiver* or care giver* or consultant* or assistant* or staff or visit* or midwife or midwives or provider* or practitioner*)).tw,kw. | 29630 |
| 10 | paramedical personnel/ | 16681 |
| 11 | health auxiliary/ | 9820 |
| 12 | (paraprofessional* or paramedic* or allied health personnel or allied health worker* or support worker* or home health aide*).tw,kw. | 19561 |
| 13 | (trained adj3 (volunteer* or health worker* or mother*)).tw,kw. | 3324 |
| 14 | ((communit* or village* or frontline*) adj3 (health worker* or health care worker* or healthcare worker* or distributor* or worker* or provider*)).tw,kw. | 21315 |
| 15 | (communit* adj3 (volunteer* or aide* or support)).tw,kw. | 15857 |
| 16 | ((birth or childbirth or labor or labour) adj1 (attendant* or assistant*)).tw,kw. | 2900 |
| 17 | montrice*.tw,kw. | 0 |
| 18 | ((lay or peer) adj1 (volunteer* or mentor* or counsel* or support or intervention*)).tw,kw. | 12868 |
| 19 | (church based adj3 (intervention* or program* or counsel*)).tw,kw. | 230 |
| 20 | (linkworker* or link worker*).tw,kw. | 192 |
| 21 | barefoot doctor*.tw,kw. | 147 |
| 22 | ((health or healthcare) adj1 outreach).tw,kw. | 725 |
| 23 | Home Care/ | 72572 |
| 24 | (home adj1 (care or aide* or nursing or support or intervention* or treatment* or visit*)).tw,kw. | 79808 |
| 25 | ((care or aide* or nursing or support or intervention* or treatment* or visit*) adj3 (lay or volunteer* or voluntary)).tw,kw. | 9150 |
| 26 | (Auxiliary adj3 (worker* or nurse* or midwives or midwife)).tw,kw. | 854 |
| 27 | (expert patient* or health promoter* or health extension worker* or mentor mother*).tw,kw. | 2112 |
| 28 | or/1-27 | 310970 |
| 29 | Economics/ | 247224 |
| 30 | Cost/ | 66138 |
| 31 | exp Health Economics/ | 1051128 |
| 32 | exp Budgets/ | 34387 |
| 33 | budget*.ti,ab,kf. | 48974 |
| 34 | (economic* or cost or costs or costly or costing or price or prices or pricing or expenditure or expenditures or expense or expenses or financial or finance or finances or financed).ti,kf. | 355771 |
| 35 | (economic* or cost or costs or costly or costing or price or prices or pricing or expenditure or expenditures or expense or expenses or financial or finance or finances or financed).ab. /freq=2 | 542091 |
| 36 | (cost* adj2 (effective* or utilit* or benefit* or minimi* or analy* or outcome or outcomes)).ab,kf. | 295373 |
| 37 | (value adj2 (money or monetary)).ti,ab,kf. | 4170 |
| 38 | exp economic model/ | 3794 |
| 39 | economic model*.ab,kf. | 6345 |
| 40 | Decision Theory/ | 1887 |
| 41 | Decision Tree/ | 21876 |
| 42 | (decision* adj2 (tree* or analy* or model*)).ti,ab,kf. | 52577 |
| 43 | Disability-Adjusted Life Year/ | 4550 |
| 44 | (Disability-Adjusted Life Years or DALYS).tw,kw. | 6446 |
| 45 | quality-adjusted life year/ | 35690 |
| 46 | (quality-adjusted life years or QALYS).tw,kw. | 23305 |
| 47 | Return on investment.tw,kw. | 3276 |
| 48 | (multiple criteria decision analysis or MCDA).tw,kw. | 1534 |
| 49 | cost benefit analysis/ | 94545 |
| 50 | cost-benefit analysis.tw,kw. | 8114 |
| 51 | (Economic evaluation* or economic analys*).tw,kw. | 33888 |
| 52 | Financial management/ | 125641 |
| 53 | (Financial planning or financial management).tw,kw. | 2268 |
| 54 | or/29-53 | 1768310 |
| **APA PsycInfo (1806 to July Week 1 2023)** | | |
| 1 | volunteers/ | 6011 |
| 2 | health auxiliar*.tw. | 1 |
| 3 | peer group*.tw. | 6619 |
| 4 | health visitor*.tw. | 534 |
| 5 | doula*.tw. | 186 |
| 6 | douladural*.tw. | 0 |
| 7 | ((lay or voluntary or volunteer* or untrained or unlicensed or non-professional* or nonprofessional* or informal or non-formal) adj5 (worker* or visitor* or attendant* or aide* or support* or person* or helper* or carer* or caregiver* or care giver* or consultant* or assistant* or staff or visit* or midwife or midwives or provider* or practitioner*)).tw. | 16010 |
| 8 | Allied Health Personnel/ | 1351 |
| 9 | paramedics/ | 440 |
| 10 | (paraprofessional* or paramedic* or allied health personnel or allied health worker* or support worker* or home health aide*).tw. | 5058 |
| 11 | (trained adj3 (volunteer* or health worker* or mother*)).tw. | 751 |
| 12 | ((communit* or village* or frontline*) adj3 (health worker* or health care worker* or healthcare worker* or distributor* or worker* or provider*)).tw. | 6487 |
| 13 | (communit* adj3 (volunteer* or aide* or support)).tw. | 9618 |
| 14 | ((birth or childbirth or labor or labour) adj1 (attendant* or assistant*)).tw. | 300 |
| 15 | montrice*.tw. | 0 |
| 16 | ((lay or peer) adj1 (volunteer* or mentor* or counsel* or support or intervention*)).tw. | 8801 |
| 17 | (church based adj3 (intervention* or program* or counsel*)).tw. | 207 |
| 18 | (linkworker* or link worker*).tw. | 64 |
| 19 | barefoot doctor*.tw. | 8 |
| 20 | ((health or healthcare) adj1 outreach).tw. | 282 |
| 21 | Home Care/ | 7596 |
| 22 | (home adj1 (care or aide* or nursing or support or intervention* or treatment* or visit*)).tw. | 24703 |
| 23 | ((care or aide* or nursing or support or intervention* or treatment* or visit*) adj3 (lay or volunteer* or voluntary)).tw. | 3081 |
| 24 | (Auxiliary adj3 (worker* or nurse* or midwives or midwife)).tw. | 131 |
| 25 | (expert patient* or health promoter* or health extension worker* or mentor mother*).tw. | 465 |
| 26 | or/1-25 | 84206 |
| 27 | Economics/ | 27668 |
| 28 | exp "Costs and Cost Analysis"/ | 49131 |
| 29 | Health Care Economics/ | 1183 |
| 30 | exp Budgets/ | 1427 |
| 31 | budget*.tw. | 10217 |
| 32 | (economic* or cost or costs or costly or costing or price or prices or pricing or expenditure or expenditures or expense or expenses or financial or finance or finances or financed).ti. | 46803 |
| 33 | (economic* or cost or costs or costly or costing or price or prices or pricing or expenditure or expenditures or expense or expenses or financial or finance or finances or financed).ab. /freq=2 | 105796 |
| 34 | (cost* adj2 (effective* or utilit* or benefit* or minimi* or analy* or outcome or outcomes)).ab. | 28207 |
| 35 | (value adj2 (money or monetary)).tw. | 1154 |
| 36 | economic model*.ab. | 1090 |
| 37 | exp Decision Theory/ | 1852 |
| 38 | (decision* adj2 (tree* or analy* or model*)).tw. | 11140 |
| 39 | (Disability-Adjusted Life Years or DALYS).tw. | 539 |
| 40 | (quality-adjusted life years or QALYS).tw. | 1386 |
| 41 | Return on investment.tw. | 1050 |
| 42 | (multiple criteria decision analysis or MCDA).tw. | 125 |
| 43 | cost-benefit analysis.tw. | 1207 |
| 44 | (Economic evaluation* or economic analys*).tw. | 3545 |
| 45 | (Financial planning or financial management).tw. | 1195 |
| 46 | or/27-45 | 184947 |
| **Global Health (1910 to 2023 Week 26)** | | |
| 1 | Volunteers/ | 2600 |
| 2 | health auxiliar*.ti,ab. | 56 |
| 3 | peer group*.ti,ab. | 677 |
| 4 | health visitor*.ti,ab. | 1154 |
| 5 | doula*.ti,ab. | 81 |
| 6 | douladural*.ti,ab. | 0 |
| 7 | ((lay or voluntary or volunteer* or untrained or unlicensed or non-professional* or nonprofessional* or informal or non-formal) adj5 (worker* or visitor* or attendant* or aide* or support* or person* or helper* or carer* or caregiver* or care giver* or consultant* or assistant* or staff or visit* or midwife or midwives or provider* or practitioner*)).ti,ab. | 5562 |
| 8 | Community Health Services/ | 5894 |
| 9 | (paraprofessional* or paramedic* or allied health personnel or allied health worker* or support worker* or home health aide*).ti,ab. | 1994 |
| 10 | (trained adj3 (volunteer* or health worker* or mother*)).ti,ab. | 1133 |
| 11 | ((communit* or village* or frontline*) adj3 (health worker* or health care worker* or healthcare worker* or distributor* or worker* or provider*)).ti,ab. | 8853 |
| 12 | (communit* adj3 (volunteer* or aide* or support)).ti,ab. | 4050 |
| 13 | ((birth or childbirth or labor or labour) adj1 (attendant* or assistant*)).ti,ab. | 1780 |
| 14 | montrice*.ti,ab. | 0 |
| 15 | ((lay or peer) adj1 (volunteer* or mentor* or counsel* or support or intervention*)).ti,ab. | 1930 |
| 16 | (church based adj3 (intervention* or program* or counsel*)).ti,ab. | 70 |
| 17 | (linkworker* or link worker*).ti,ab. | 55 |
| 18 | barefoot doctor*.ti,ab. | 70 |
| 19 | ((health or healthcare) adj1 outreach).ti,ab. | 213 |
| 20 | Home Care/ | 2644 |
| 21 | (home adj1 (care or aide* or nursing or support or intervention* or treatment* or visit*)).ti,ab. | 9497 |
| 22 | ((care or aide* or nursing or support or intervention* or treatment* or visit*) adj3 (lay or volunteer* or voluntary)).ti,ab. | 1604 |
| 23 | (Auxiliary adj3 (worker* or nurse* or midwives or midwife)).ti,ab. | 417 |
| 24 | (expert patient* or health promoter* or health extension worker* or mentor mother*).ti,ab. | 979 |
| 25 | or/1-24 | 41978 |
| 26 | Economics/ | 17725 |
| 27 | exp Costs/ | 37464 |
| 28 | exp Economic Analysis/ | 20481 |
| 29 | Budgets/ | 675 |
| 30 | budget*.ti,ab. | 8320 |
| 31 | (economic* or cost or costs or costly or costing or price or prices or pricing or expenditure or expenditures or expense or expenses or financial or finance or finances or financed).ti. | 50103 |
| 32 | (economic* or cost or costs or costly or costing or price or prices or pricing or expenditure or expenditures or expense or expenses or financial or finance or finances or financed).ab. /freq=2 | 109726 |
| 33 | (cost* adj2 (effective* or utilit* or benefit* or minimi* or analy* or outcome or outcomes)).ab. | 40832 |
| 34 | (value adj2 (money or monetary)).ti,ab. | 988 |
| 35 | economic model*.ab. | 736 |
| 36 | (decision* adj2 (tree* or analy* or model*)).ti,ab. | 5165 |
| 37 | (Disability-Adjusted Life Years or DALYS).ti,ab. | 2998 |
| 38 | (quality-adjusted life years or QALYS).ti,ab. | 2117 |
| 39 | Return on investment.ti,ab. | 553 |
| 40 | (multiple criteria decision analysis or MCDA).ti,ab. | 233 |
| 41 | cost-benefit analysis.ti,ab. | 1198 |
| 42 | (Economic evaluation* or economic analys*).ti,ab. | 4589 |
| 43 | (Financial planning or financial management).ti,ab. | 299 |
| 44 | or/26-43  (Afghanistan or Burundi or Burkina Faso or Central African Republic or Congo or Eritrea or Ethiopia or Guinea or Gambia or Guinea-Bissau or Liberia or Madagascar or Mali or Mozambique or Malawi or Niger or North Korea or Rwanda or Sudan or Sierra Leone or Somalia or South Sudan or Syrian Arab Republic or Chad or Togo or Uganda or Yemen or Zambia or Angola or Benin or Bangladesh or Bolivia or Bhutan or "cote d'ivoir*" or "cote d' ivoir*" or cote divoir* or cote d ivoir* or ivory coast* or Cameroon or Comoros or Cabo Verde or Djibouti or Algeria or Egypt or Micronesia or Ghana or Honduras or Haiti or Indonesia or India or Iran or Kenya or Kyrgyzstan or Cambodia or Kiribati or Laos or Lebanon or Sri Lanka or Lesotho or Morocco or Myanmar or Burma or Mongolia or Mauritania or Nigeria or Nicaragua or Nepal or Pakistan or Philippines or Papua New Guinea or "West Bank" or Gaza or Senegal or Solomon Islands or El Salvador or "Sao Tome and Principe" or Eswatini or Swaziland or Tajikistan or Timor-Leste or Tunisia or Tanzania or Ukraine or Uzbekistan or Vietnam or Vanuatu or Samoa or Zimbabwe or Afghan* or Burundian* or Burkinabe* or Central African* or Congolese or Eritrean* or Ethiopian* or Guinean* or Gambian* or Guinea-Bissauan* or Liberian* or Malagasy* or Malian* or Mozambican* or Malawian* or Nigerien* or North Korean* or Rwandan* or Sudanese or Sierra Leonean* or Somali* or Syrian or Chadian* or Togolese or Ugandan* or Yemeni* or Zambian* or Angolan* or Benin* or Bangladeshi* or Bolivian* or Bhutanese* or Ivorian* or Cameroonian* or Comorian* or Cape Verdean* or Djiboutian* or Algerian* or Egyptian* or Micronesian* or Ghanaian* or Honduran* or Haitian* or Indonesian* or (Indian* not indiana) or Iranian* or Kenyan* or Kyrgyz* or kirgiz* or kirghiz* or Cambodian* or Kiribatian* or Laotian* or Lebanese or Sri Lankan* or Basotho* or Moroccan* or Burmese or Mongolian* or Mauritanian* or Nigerian* or Nicaraguan* or Nepalese or Pakistani* or Filipino* or Papua New Guinean* or Palestinian* or Senegalese or Solomon Islander* or Salvadoran* or Salvadorian* or Sao Tomean* or Eswatini* or swazi* or swati* or Tajik* or Timorese or Tunisian* or Tanzanian* or Ukrainian* or Uzbek* or Vietnamese or Vanuatuan* or Samoan* or Zimbabwean* or africa* or arab* countr* or middle east* or global south or sahara* or subsahara* or magreb* or maghrib* or west indies* or caribbean* or central america* or latin america* or south america* or central asia* or north asia* or northern asia* or southeastern asia* or south eastern asia* or southeast asia* or south east asia* or west asia* or western asia* or east europe* or eastern europe* or developing countr* or developing nation* or developing population* or developing world or less developed countr* or less developed nation* or less developed world or lesser developed countr* or lesser developed nation* or lesser developed world or under developed countr* or under developed nation* or under developed world or underdeveloped countr* or underdeveloped nation* or underdeveloped world or middle income countr* or middle income nation* or middle income population* or low income countr* or low income nation* or low income population* or lower income countr* or lower income nation* or lower income population* or underserved countr* or underserved nation* or underserved population* or under served population* or under served nation* or under served population* or deprived countr* or deprived population* or high burden countr* or high burden nation* or countdown countr* or countdown nation* or poor countr* or poor nation* or poor population* or poor world or poorer countr* or poorer nation* or poorer population* or poorer world or developing econom* or less developed econom* or underdeveloped econom* or under developed econom* or middle income econom* or low income econom* or lower income econom* or low gdp or low gnp or low gross domestic or low gross national or lower gdp or lower gnp or lower gross domestic or lower gross national or lmic or lmics or third world or lami countr* or transitional countr* or emerging econom* or emerging nation*).ti,ab,hw. | 173278 |
| 46 | 25 and 44 and 45 | 2661 |
| 47 | limit 46 to yr="2015 -Current" | 1434 |
| **AMED (Allied and Complementary Medicine) (1985 to June 2023)** | | |
| 1 | Voluntary Workers/ | 206 |
| 2 | Hospital Volunteers/ | 13 |
| 3 | health auxiliar*.ti,ab. | 0 |
| 4 | peer group*.ti,ab. | 56 |
| 5 | health visitor*.ti,ab. | 29 |
| 6 | doula*.ti,ab. | 7 |
| 7 | douladural*.ti,ab. | 0 |
| 8 | ((lay or voluntary or volunteer* or untrained or unlicensed or non-professional* or nonprofessional* or informal or non-formal) adj5 (worker* or visitor* or attendant* or aide* or support* or person* or helper* or carer* or caregiver* or care giver* or consultant* or assistant* or staff or visit* or midwife or midwives or provider* or practitioner*)).ti,ab. | 1017 |
| 9 | Allied Health Personnel/ | 691 |
| 10 | Community Health Services/ | 2165 |
| 11 | (paraprofessional* or paramedic* or allied health personnel or allied health worker* or support worker* or home health aide*).ti,ab. | 258 |
| 12 | (trained adj3 (volunteer* or health worker* or mother*)).ti,ab. | 49 |
| 13 | ((communit* or village* or frontline*) adj3 (health worker* or health care worker* or healthcare worker* or distributor* or worker* or provider*)).ti,ab. | 242 |
| 14 | (communit* adj3 (volunteer* or aide* or support)).ti,ab. | 553 |
| 15 | ((birth or childbirth or labor or labour) adj1 (attendant* or assistant*)).ti,ab. | 6 |
| 16 | montrice*.ti,ab. | 0 |
| 17 | ((lay or peer) adj1 (volunteer* or mentor* or counsel* or support or intervention*)).ti,ab. | 348 |
| 18 | (church based adj3 (intervention* or program* or counsel*)).ti,ab. | 3 |
| 19 | (linkworker* or link worker*).ti,ab. | 2 |
| 20 | barefoot doctor*.ti,ab. | 3 |
| 21 | ((health or healthcare) adj1 outreach).ti,ab. | 9 |
| 22 | Home Care Services/ | 1994 |
| 23 | (home adj1 (care or aide* or nursing or support or intervention* or treatment* or visit*)).ti,ab. | 2889 |
| 24 | ((care or aide* or nursing or support or intervention* or treatment* or visit*) adj3 (lay or volunteer* or voluntary)).ti,ab. | 468 |
| 25 | (Auxiliary adj3 (worker* or nurse* or midwives or midwife)).ti,ab. | 5 |
| 26 | (expert patient* or health promoter* or health extension worker* or mentor mother*).ti,ab. | 27 |
| 27 | or/1-26 | 9229 |
| 28 | Economics/ | 4859 |
| 29 | exp "Costs and Cost Analysis"/ | 1560 |
| 30 | budget*.ti,ab. | 223 |
| 31 | (economic* or cost or costs or costly or costing or price or prices or pricing or expenditure or expenditures or expense or expenses or financial or finance or finances or financed).ti. | 2504 |
| 32 | (economic* or cost or costs or costly or costing or price or prices or pricing or expenditure or expenditures or expense or expenses or financial or finance or finances or financed).ab. /freq=2 | 3305 |
| 33 | (cost* adj2 (effective* or utilit* or benefit* or minimi* or analy* or outcome or outcomes)).ab. | 2076 |
| 34 | (value adj2 (money or monetary)).ti,ab. | 21 |
| 35 | economic model*.ab. | 7 |
| 36 | (decision* adj2 (tree* or analy* or model*)).ti,ab. | 260 |
| 37 | (Disability-Adjusted Life Years or DALYS).ti,ab. | 19 |
| 38 | (quality-adjusted life years or QALYS).ti,ab. | 109 |
| 39 | Return on investment.ti,ab. | 13 |
| 40 | (multiple criteria decision analysis or MCDA).ti,ab. | 1 |
| 41 | Cost-Benefit Analysis/ | 698 |
| 42 | cost-benefit analysis.ti,ab. | 48 |
| 43 | (Economic evaluation* or economic analys*).ti,ab. | 214 |
| 44 | Financial management/ | 132 |
| 45 | (Financial planning or financial management).ti,ab. | 42 |
| 46 | or/28-45  Afghanistan or Burundi or Burkina Faso or Central African Republic or Congo or Eritrea or Ethiopia or Guinea or Gambia or Guinea-Bissau or Liberia or Madagascar or Mali or Mozambique or Malawi or Niger or North Korea or Rwanda or Sudan or Sierra Leone or Somalia or South Sudan or Syrian Arab Republic or Chad or Togo or Uganda or Yemen or Zambia or Angola or Benin or Bangladesh or Bolivia or Bhutan or "cote d'ivoir*" or "cote d' ivoir*" or cote divoir* or cote d ivoir* or ivory coast* or Cameroon or Comoros or Cabo Verde or Djibouti or Algeria or Egypt or Micronesia or Ghana or Honduras or Haiti or Indonesia or India or Iran or Kenya or Kyrgyzstan or Cambodia or Kiribati or Laos or Lebanon or Sri Lanka or Lesotho or Morocco or Myanmar or Burma or Mongolia or Mauritania or Nigeria or Nicaragua or Nepal or Pakistan or Philippines or Papua New Guinea or "West Bank" or Gaza or Senegal or Solomon Islands or El Salvador or "Sao Tome and Principe" or Eswatini or Swaziland or Tajikistan or Timor-Leste or Tunisia or Tanzania or Ukraine or Uzbekistan or Vietnam or Vanuatu or Samoa or Zimbabwe or Afghan* or Burundian* or Burkinabe* or Central African* or Congolese or Eritrean* or Ethiopian* or Guinean* or Gambian* or Guinea-Bissauan* or Liberian* or Malagasy* or Malian* or Mozambican* or Malawian* or Nigerien* or North Korean* or Rwandan* or Sudanese or Sierra Leonean* or Somali* or Syrian or Chadian* or Togolese or Ugandan* or Yemeni* or Zambian* or Angolan* or Benin* or Bangladeshi* or Bolivian* or Bhutanese* or Ivorian* or Cameroonian* or Comorian* or Cape Verdean* or Djiboutian* or Algerian* or Egyptian* or Micronesian* or Ghanaian* or Honduran* or Haitian* or Indonesian* or (Indian* not indiana) or Iranian* or Kenyan* or Kyrgyz* or kirgiz* or kirghiz* or Cambodian* or Kiribatian* or Laotian* or Lebanese or Sri Lankan* or Basotho* or Moroccan* or Burmese or Mongolian* or Mauritanian* or Nigerian* or Nicaraguan* or Nepalese or Pakistani* or Filipino* or Papua New Guinean* or Palestinian* or Senegalese or Solomon Islander* or Salvadoran* or Salvadorian* or Sao Tomean* or Eswatini* or swazi* or swati* or Tajik* or Timorese or Tunisian* or Tanzanian* or Ukrainian* or Uzbek* or Vietnamese or Vanuatuan* or Samoan* or Zimbabwean* or africa* or arab* countr* or middle east* or global south or sahara* or subsahara* or magreb* or maghrib* or west indies* or caribbean* or central america* or latin america* or south america* or central asia* or north asia* or northern asia* or southeastern asia* or south eastern asia* or southeast asia* or south east asia* or west asia* or western asia* or east europe* or eastern europe* or developing countr* or developing nation* or developing population* or developing world or less developed countr* or less developed nation* or less developed world or lesser developed countr* or lesser developed nation* or lesser developed world or under developed countr* or under developed nation* or under developed world or underdeveloped countr* or underdeveloped nation* or underdeveloped world or middle income countr* or middle income nation* or middle income population* or low income countr* or low income nation* or low income population* or lower income countr* or lower income nation* or lower income population* or underserved countr* or underserved nation* or underserved population* or under served population* or under served nation* or under served population* or deprived countr* or deprived population* or high burden countr* or high burden nation* or countdown countr* or countdown nation* or poor countr* or poor nation* or poor population* or poor world or poorer countr* or poorer nation* or poorer population* or poorer world or developing econom* or less developed econom* or underdeveloped econom* or under developed econom* or middle income econom* or low income econom* or lower income econom* or low gdp or low gnp or low gross domestic or low gross national or lower gdp or lower gnp or lower gross domestic or lower gross national or lmic or lmics or third world or lami countr* or transitional countr* or emerging econom* or emerging nation*).ti,ab,hw. | 9614 |
| 48 | 27 and 46 and 47 | 39 |
| 49 | limit 48 to yr="2015 -Current" | 14 |
| Cochrane Central Register of Controlled Trials | | |
| ID | Search | Hits |
| #1 | MeSH descriptor: [Volunteers] this term only | 382 |
| #2 | MeSH descriptor: [Hospital Volunteers] this term only | 3 |
| #3 | (health auxiliar*):ti,ab,kw | 1044 |
| #4 | (peer group*):ti,ab,kw | 9120 |
| #5 | (health visitor*):ti,ab,kw | 606 |
| #6 | MeSH descriptor: [Doulas] this term only | 12 |
| #7 | (doula* OR douladural*):ti,ab,kw | 94 |
| #8 | ((lay OR voluntary OR volunteer* OR untrained OR unlicensed OR non-professional* OR nonprofessional* OR informal OR non-formal) NEAR/5 (worker* OR visitor* OR attendant* OR aide* OR support* OR person* OR helper* OR carer* OR caregiver* OR care giver* OR consultant* OR assistant* OR staff OR visit* OR midwife OR midwives OR provider* OR practitioner*)):ti,ab,kw | 4590 |
| #9 | MeSH descriptor: [Allied Health Personnel] this term only | 343 |
| #10 | MeSH descriptor: [Community Health Workers] this term only | 706 |
| #11 | MeSH descriptor: [Paramedics] this term only | 3 |
| #12 | (paraprofessional* OR paramedic* OR allied health personnel OR allied health worker* OR support worker* OR home health aide*):ti,ab,kw | 5104 |
| #13 | (trained NEAR/3 (volunteer* OR health worker* OR mother*)):ti,ab,kw | 1916 |
| #14 | ((communit* OR village* OR frontline*) NEAR/3 (health worker* OR health care worker* OR healthcare worker* OR distributor* OR worker* OR provider*)):ti,ab,kw | 13934 |
| #15 | (communit* NEAR/3 (volunteer* OR aide* OR support)):ti,ab,kw | 1540 |
| #16 | ((birth OR childbirth OR labor OR labour) NEAR/1 (attendant* OR assistant*)):ti,ab,kw | 289 |
| #17 | (montrice*):ti,ab,kw | 0 |
| #18 | ((lay OR peer) NEAR/1 (volunteer* OR mentor* OR counsel* OR support OR intervention*)):ti,ab,kw | 2622 |
| #19 | (church based NEAR/3 (intervention* OR program* OR counsel*)):ti,ab,kw | 174 |
| #20 | (linkworker* OR link worker*):ti,ab,kw | 262 |
| #21 | (barefoot doctor*):ti,ab,kw | 4 |
| #22 | ((health OR healthcare) NEAR/1 outreach):ti,ab,kw | 58 |
| #23 | MeSH descriptor: [Home Care Services] this term only | 2155 |
| #24 | (home NEAR/1 (care OR aide* OR nursing OR support OR intervention* OR treatment* OR visit*)):ti,ab,kw | 15859 |
| #25 | ((care OR aide* OR nursing OR support OR intervention* OR treatment* OR visit*) NEAR/3 (lay OR volunteer* OR voluntary)):ti,ab,kw | 3363 |
| #26 | (Auxiliary NEAR/3 (worker* OR nurse* OR midwives OR midwife)):ti,ab,kw | 62 |
| #27 | (expert patient* OR health promoter* OR health extension worker* OR mentor mother*):ti,ab,kw | 7172 |
| #28 | {OR #1-#27} | 55315 |
| #29 | MeSH descriptor: [Economics] this term only | 84 |
| #30 | MeSH descriptor: [Costs and Cost Analysis] explode all trees | 14572 |
| #31 | MeSH descriptor: [Economics, Nursing] this term only | 13 |
| #32 | MeSH descriptor: [Economics, Medical] this term only | 32 |
| #33 | MeSH descriptor: [Fees and Charges] explode all trees | 321 |
| #34 | MeSH descriptor: [Budgets] explode all trees | 56 |
| #35 | (budget*):ti,ab,kw | 1466 |
| #36 | (economic* OR cost OR costs OR costly OR costing OR price OR prices OR pricing OR expenditure OR expenditures OR expense OR expenses OR financial OR finance OR finances OR financed):ti,kw | 44528 |
| #37 | (cost* NEAR/2 (effective* OR utilit* OR benefit* OR minimi* OR analy* OR outcome OR outcomes)):ab,kw | 40612 |
| #38 | (value NEAR/2 (money OR monetary)):ti,ab,kw | 369 |
| #39 | MeSH descriptor: [Models, Economic] explode all trees | 571 |
| #40 | (economic model*):ti,ab,kw | 4999 |
| #41 | MeSH descriptor: [Decision Theory] explode all trees | 339 |
| #42 | (decision* NEAR/2 (tree* OR analy* OR model*)):ti,ab,kw | 3202 |
| #43 | MeSH descriptor: [Disability-Adjusted Life Years] this term only | 3 |
| #44 | (Disability-Adjusted Life Years OR DALYS):ti,ab,kw | 279 |
| #45 | MeSH descriptor: [Quality-Adjusted Life Years] this term only | 1940 |
| #46 | (quality-adjusted life years OR QALYS):ti,ab,kw | 5866 |
| #47 | (Return on investment):ti,ab,kw | 223 |
| #48 | (multiple criteria decision analysis OR MCDA):ti,ab,kw | 462 |
| #49 | MeSH descriptor: [Cost-Benefit Analysis] this term only | 9824 |
| #50 | (cost-benefit analysis):ti,ab,kw | 12281 |
| #51 | (Economic evaluation* OR economic analys*):ti,ab,kw | 14683 |
| #52 | MeSH descriptor: [Financial Management] this term only | 95 |
| #53 | (Financial planning OR financial management):ti,ab,kw | 2613 |
| #54 | {OR #29-#53}  (Afghanistan OR Burundi OR Burkina Faso OR Central African Republic OR Congo OR Eritrea OR Ethiopia OR Guinea OR Gambia OR Guinea-Bissau OR Liberia OR Madagascar OR Mali OR Mozambique OR Malawi OR Niger OR North Korea OR Rwanda OR Sudan OR Sierra Leone OR Somalia OR South Sudan OR Syrian Arab Republic OR Chad OR Togo OR Uganda OR Yemen OR Zambia OR Angola OR Benin OR Bangladesh OR Bolivia OR Bhutan OR cote d'ivoir* OR cote d' ivoir* OR cote divoir* OR cote d ivoir* OR ivory coast* OR Cameroon OR Comoros OR Cabo Verde OR Djibouti OR Algeria OR Egypt OR Micronesia OR Ghana OR Honduras OR Haiti OR Indonesia OR India OR Iran OR Kenya OR Kyrgyzstan OR Cambodia OR Kiribati OR Laos OR Lebanon OR Sri Lanka OR Lesotho OR Morocco OR Myanmar OR Burma OR Mongolia OR Mauritania OR Nigeria OR Nicaragua OR Nepal OR Pakistan OR Philippines OR Papua New Guinea OR "West Bank" OR Gaza OR Senegal OR Solomon Islands OR El Salvador OR "Sao Tome and Principe" OR Eswatini OR Swaziland OR Tajikistan OR Timor-Leste OR Tunisia OR Tanzania OR Ukraine OR Uzbekistan OR Vietnam OR Vanuatu OR Samoa OR Zimbabwe OR Afghan* OR Burundian* OR Burkinabe* OR Central African* OR Congolese OR Eritrean* OR Ethiopian* OR Guinean* OR Gambian* OR Guinea-Bissauan* OR Liberian* OR Malagasy* OR Malian* OR Mozambican* OR Malawian* OR Nigerien* OR North Korean* OR Rwandan* OR Sudanese OR Sierra Leonean* OR Somali* OR Syrian OR Chadian* OR Togolese OR Ugandan* OR Yemeni* OR Zambian* OR Angolan* OR Benin* OR Bangladeshi* OR Bolivian* OR Bhutanese* OR Ivorian* OR Cameroonian* OR Comorian* OR Cape Verdean* OR Djiboutian* OR Algerian* OR Egyptian* OR Micronesian* OR Ghanaian* OR Honduran* OR Haitian* OR Indonesian* OR (Indian* not indiana) OR Iranian* OR Kenyan* OR Kyrgyz* OR kirgiz* OR kirghiz* OR Cambodian* OR Kiribatian* OR Laotian* OR Lebanese OR Sri Lankan* OR Basotho* OR Moroccan* OR Burmese OR Mongolian* OR Mauritanian* OR Nigerian* OR Nicaraguan* OR Nepalese OR Pakistani* OR Filipino* OR Papua New Guinean* OR Palestinian* OR Senegalese OR Solomon Islander* OR Salvadoran* OR Salvadorian* OR Sao Tomean* OR Eswatini* OR swazi* OR swati* OR Tajik* OR Timorese OR Tunisian* OR Tanzanian* OR Ukrainian* OR Uzbek* OR Vietnamese OR Vanuatuan* OR Samoan* OR Zimbabwean* OR africa* OR arab* countr* OR middle east* OR global south OR sahara* OR subsahara* OR magreb* OR maghrib* OR west indies* OR caribbean* OR central america* OR latin america* OR south america* OR central asia* OR north asia* OR northern asia* OR southeastern asia* OR south eastern asia* OR southeast asia* OR south east asia* OR west asia* OR western asia* OR east europe* OR eastern europe* OR developing countr* OR developing nation* OR developing population* OR developing world OR less developed countr* OR less developed nation* OR less developed world OR lesser developed countr* OR lesser developed nation* OR lesser developed world OR under developed countr* OR under developed nation* OR under developed world OR underdeveloped countr* OR underdeveloped nation* OR underdeveloped world OR middle income countr* OR middle income nation* OR middle income population* OR low income countr* OR low income nation* OR low income population* OR lower income countr* OR lower income nation* OR lower income population* OR underserved countr* OR underserved nation* OR underserved population* OR under served population* OR under served nation* OR under served population* OR deprived countr* OR deprived population* OR high burden countr* OR high burden nation* OR countdown countr* OR countdown nation* OR poor countr* OR poor nation* OR poor population* OR poor world OR poorer countr* OR poorer nation* OR poorer population* OR poorer world OR developing econom* OR less developed econom* OR underdeveloped econom* OR under developed econom* OR middle income econom* OR low income econom* OR lower income econom* OR low gdp OR low gnp OR low gross domestic OR low gross national OR lower gdp OR lower gnp OR lower gross domestic OR lower gross national OR lmic OR lmics OR third world OR lami countr* OR transitional countr* OR emerging econom* OR emerging nation*):ti,ab,kw | 70292 |
| #56 | #28 AND #54 AND #55 with Publication Year from 2015 to 2023, in Trials | 1550 |
| **CINAHL** | | |
| S1 | MH Volunteers OR MH Hospital Volunteers OR TI ( “Health auxiliar*” OR “peer group*” OR “health visitor*” OR doula* OR “Allied health personnel” OR “Community health worker*” OR paramedic* OR paraprofessional* “allied health worker*” OR “support worker*” OR “support worker*” OR “home health aide*” OR montrice* OR linkworker* OR “link worker*” OR “barefoot doctor*” OR “expert patient*” OR “health promoter*” OR “health extension worker*” OR “mentor mother*” ) OR AB ( “Health auxiliar*” OR “peer group*” OR “health visitor*” OR doula* OR “Allied health personnel” OR “Community health worker*” OR paramedic* OR paraprofessional* “allied health worker*” OR “support worker*” OR “support worker*” OR “home health aide*” OR montrice* OR linkworker* OR “link worker*” OR “barefoot doctor*” OR “expert patient*” OR “health promoter*” OR “health extension worker*” OR “mentor mother*” ) OR TI ( (lay OR voluntary OR volunteer* OR untrained OR unlicensed OR non-professional* OR nonprofessional* OR informal OR non-formal) N5 (worker* OR visitor* OR attendant* OR aide* OR support* OR person* OR helper* OR carer* OR caregiver* OR care giver* OR consultant* OR assistant* OR staff OR visit* OR midwife OR midwives OR provider* OR practitioner*) ) OR AB ( (lay OR voluntary OR volunteer* OR untrained OR unlicensed OR non-professional* OR nonprofessional* OR informal OR non-formal) N5 (worker* OR visitor* OR attendant* OR aide* OR support* OR person* OR helper* OR carer* OR caregiver* OR care giver* OR consultant* OR assistant* OR staff OR visit* OR midwife OR midwives OR provider* OR practitioner*) ) OR TI ( trained N3 (volunteer* OR “health worker*” OR mother*) ) OR AB ( trained N3 (volunteer* OR “health worker*” OR mother*) ) OR TI ( (communit* OR village* OR frontline*) N3 (“health worker*” OR “health care worker*” OR “healthcare worker*” OR distributor* OR worker* OR provider*) ) OR AB ( (communit* OR village* OR frontline*) N3 (“health worker*” OR “health care worker*” OR “healthcare worker*” OR distributor* OR worker* OR provider*) ) OR TI ( communit* N3 (volunteer* OR aide* OR support) ) OR AB ( communit* N3 (volunteer* OR aide* OR support) ) | 51,954 |
| S2 | TI ( (birth OR childbirth OR labor OR labour) N1 (attendant* OR assistant*) ) OR AB ( (birth OR childbirth OR labor OR labour) N1 (attendant* OR assistant*) ) OR TI ( (lay OR peer) N1 (volunteer* OR mentor* OR counsel* OR support OR intervention*) ) OR AB ( (lay OR peer) N1 (volunteer* OR mentor* OR counsel* OR support OR intervention*) ) OR TI ( (“church based” N3 (intervention* OR program* OR counsel*)) ) OR AB ( (“church based” N3 (intervention* OR program* OR counsel*)) ) OR TI ( ((health OR healthcare) N1 outreach) ) OR AB ( ((health OR healthcare) N1 outreach) ) OR TI ( (home N1 (care OR aide* OR nursing OR support OR intervention* OR treatment* OR visit*)) ) OR AB ( (home N1 (care OR aide* OR nursing OR support OR intervention* OR treatment* OR visit*)) ) OR TI ( ((care OR aide* OR nursing OR support OR intervention* OR treatment* OR visit*) N3 (lay OR volunteer* OR voluntary)) ) OR AB ( ((care OR aide* OR nursing OR support OR intervention* OR treatment* OR visit*) N3 (lay OR volunteer* OR voluntary)) ) | 78,769 |
| S3 | TI ( (Auxiliary N3 (worker* OR nurse* OR midwives OR midwife)) ) AND AB ( (Auxiliary N3 (worker* OR nurse* OR midwives OR midwife)) ) OR MH Allied Health Personnel OR MH community Health Workers OR MH paramedics OR MH Home Care Services | 9,406 |
| S4 | S1 OR S2 OR S3 | 129,588 |
| S5 | MH Economics OR MH ( Costs and Cost Analysis ) OR MH Economics, Nursing OR MH Economics, Medical OR MH ( Fees and Charges ) OR MH Budgets OR TI ( economic* OR cost OR costs OR costly OR costing OR price OR prices OR pricing OR expenditure OR expenditures OR expense OR expenses OR financial OR finance OR finances OR financed ) OR AB ( cost* N2 (effective* OR utilit* OR benefit* OR minimi* OR analy* OR outcome OR outcomes) ) OR TI ( (value N2 (money OR monetary)) OR (“economic model*”) OR (decision* N2 (tree* OR analy* OR model*)) ) OR AB ( (value N2 (money OR monetary)) OR (“economic model*”) OR (decision* N2 (tree* OR analy* OR model*)) ) OR TI ( ("Disability-Adjusted Life Years" OR DALYS OR "quality-adjusted life years" OR QALYS OR “Return on investment” OR “multiple criteria decision analysis” or MCDA OR “cost-benefit analysis” OR “Economic evaluation*” OR “economic analys*” OR “Financial planning” OR “financial management”) ) OR AB ( ("Disability-Adjusted Life Years" OR DALYS OR "quality-adjusted life years" OR QALYS OR “Return on investment” OR “multiple criteria decision analysis” or MCDA OR “cost-benefit analysis” OR “Economic evaluation*” OR “economic analys*” OR “Financial planning” OR “financial management”) ) | 177,125 |
|  | R Kyrgyz* OR kirgiz* OR kirghiz* OR Cambodian* OR Kiribatian* OR Laotian* OR Lebanese OR “Sri Lankan*” OR Basotho* OR Moroccan* OR Burmese OR Mongolian* OR Mauritanian* OR Nigerian* OR Nicaraguan* OR Nepalese OR Pakistani* OR Filipino* OR “Papua New Guinean*” OR Palestinian* OR Senegalese OR “Solomon Islander*” OR Salvadoran* OR Salvadorian* OR Sao Tomean* OR Eswatini* OR swazi* OR swati* OR Tajik* OR Timorese OR Tunisian* OR Tanzanian* OR Ukrainian* OR Uzbek* OR Vietnamese OR Vanuatuan* OR Samoan* OR Zimbabwean* OR africa* OR “arab* countr*” OR “middle east*” OR “global south” OR sahara* OR subsahara* OR magreb* OR maghrib* OR “west indies*” OR caribbean* OR “central america*” OR “latin america*” OR “south america*” OR “central asia*” OR “north asia*” OR “northern asia*” OR “southeastern asia*” OR “south eastern asia*” OR “southeast asia*” OR “south east asia*” OR “west asia*” OR “western asia*” OR “east europe*” OR “eastern europe*” OR “developing countr*” OR “developing nation*” OR “developing population*” OR “developing world” OR “less developed countr*” OR “less developed nation*” OR “less developed world” OR “lesser developed countr*” OR “lesser developed nation*” OR “lesser developed world” OR “under developed countr*” OR “under developed nation*” OR “under developed world” OR “underdeveloped countr*” OR “underdeveloped nation*” OR “underdeveloped world” OR “middle income countr*” OR “middle income nation*” OR “middle income population*” OR “low income countr*” OR “low income nation*” OR “low income population*” OR “lower income countr*” OR “lower income nation*” OR “lower income population*” OR “underserved countr*” OR “underserved nation*” OR “underserved population*” OR “under served population*” OR “under served nation*” OR “deprived countr*” OR “deprived population*” OR “high burden countr*” OR “high burden nation*” OR “countdown countr*” OR “countdown nation*” OR “poor countr*” OR “poor nation*” OR “poor population*” OR “poor world” OR “poorer countr*” OR “poorer nation*” OR “poorer population*” OR “poorer world” OR “developing econom*” OR “less developed econom*” OR “underdeveloped econom*” OR “under developed econom*” OR “middle income econom*” OR “low income econom*” OR “lower income econom*” OR “low gdp” OR “low gnp” OR “low gross domestic” OR “low gross national” OR “lower gdp” OR “lower gnp” OR “lower gross domestic” OR “lower gross national” OR lmic OR lmics OR “third world” OR “lami countr*” OR “transitional countr*” OR “emerging econom*” OR “emerging nation*”) ) OR AB ( (Afghanistan OR Burundi OR “Burkina Faso” OR “Central African Republic” OR Congo OR Eritrea OR Ethiopia OR Guinea OR Gambia OR Guinea-Bissau OR Liberia OR Madagascar OR Mali OR Mozambique OR Malawi OR Niger OR “North Korea” OR Rwanda OR Sudan OR “Sierra Leone” OR Somalia OR “South Sudan” OR “Syrian Arab Republic” OR Chad OR Togo OR Uganda OR Yemen OR Zambia OR Angola OR Benin OR Bangladesh OR Bolivia OR Bhutan OR "cote d'ivoir*" OR "cote d' ivoir*" OR “cote divoir*” OR “cote d ivoir*” OR “ivory coast*” OR Cameroon OR Comoros OR “Cabo Verde” OR Djibouti OR Algeria OR Egypt OR Micronesia OR Ghana OR Honduras OR Haiti OR Indonesia OR India OR Iran OR Kenya OR Kyrgyzstan OR Cambodia OR Kiribati OR Laos OR Lebanon OR “Sri Lanka” OR Lesotho OR Morocco OR Myanmar OR Burma OR Mongolia OR Mauritania OR Nigeria OR Nicaragua OR Nepal OR Pakistan OR Philippines OR “Papua New Guinea” OR "West Bank" OR Gaza OR Senegal OR “Solomon Islands” OR “El Salvador” OR "Sao Tome and Principe" OR Eswatini OR Swaziland OR Tajikistan OR Timor-Leste OR Tunisia OR Tanzania OR Ukraine OR Uzbekistan OR Vietnam OR Vanuatu OR Samoa OR Zimbabwe OR Afghan* OR Burundian* OR Burkinabe* OR “Central African*” OR Congolese OR Eritrean* OR Ethiopian* OR Guinean* OR Gambian* OR Guinea-Bissauan* OR Liberian* OR Malagasy* OR Malian* OR Mozambican* OR Malawian* OR Nigerien* OR “North Korean*” OR Rwandan* OR Sudanese OR “Sierra Leonean*” OR Somali* OR Syrian OR Chadian* OR Togolese OR Ugandan* OR Yemeni* OR Zambian* OR Angolan* OR Benin* OR Bangladeshi* OR Bolivian* OR Bhutanese* OR Ivorian* OR Cameroonian* OR Comorian* OR Cape Verdean* OR Djiboutian* OR Algerian* OR Egyptian* OR Micronesian* OR Ghanaian* OR Honduran* OR Haitian* OR Indonesian* OR (Indian* NOT indiana) OR Iranian* OR Kenyan* OR Kyrgyz* OR kirgiz* OR kirghiz* OR Cambodian* OR Kiribatian* OR Laotian* OR Lebanese OR “Sri Lankan*” OR Basotho* OR Moroccan* OR Burmese OR Mongolian* OR Mauritanian* OR Nigerian* OR Nicaraguan* OR Nepalese OR Pakistani* OR Filipino* OR “Papua New Guinean*” OR Palestinian* OR Senegalese OR “Solomon Islander*” OR Salvadoran* OR Salvadorian* OR Sao Tomean* OR Eswatini* OR swazi* OR swati* OR Tajik* OR Timorese OR Tunisian* OR Tanzanian* OR Ukrainian* OR Uzbek* OR Vietnamese OR Vanuatuan* OR Samoan* OR Zimbabwean* OR africa* OR “arab* countr*” OR “middle east*” OR “global south” OR sahara* OR subsahara* OR magreb* OR maghrib* OR “west indies*” OR caribbean* OR “central america*” OR “latin america*” OR “south america*” OR “central asia*” OR “north asia*” OR “northern asia*” OR “southeastern asia*” OR “south eastern asia*” OR “southeast asia*” OR “south east asia*” OR “west asia*” OR “western asia*” OR “east europe*” OR “eastern europe*” OR “developing countr*” OR “developing nation*” OR “developing population*” OR “developing world” OR “less developed countr*” OR “less developed nation*” OR “less developed world” OR “lesser developed countr*” OR “lesser developed nation*” OR “lesser developed world” OR “under developed countr*” OR “under developed nation*” OR “under developed world” OR “underdeveloped countr*” OR “underdeveloped nation*” OR “underdeveloped world” OR “middle income countr*” OR “middle income nation*” OR “middle income population*” OR “low income countr*” OR “low income nation*” OR “low income population*” OR “lower income countr*” OR “lower income nation*” OR “lower income population*” OR “underserved countr*” OR “underserved nation*” OR “underserved population*” OR “under served population*” OR “under served nation*” OR “deprived countr*” OR “deprived population*” OR “high burden countr*” OR “high burden nation*” OR “countdown countr*” OR “countdown nation*” OR “poor countr*” OR “poor nation*” OR “poor population*” OR “poor world” OR “poorer countr*” OR “poorer nation*” OR “poorer population*” OR “poorer world” OR “developing econom*” OR “less developed econom*” OR “underdeveloped econom*” OR “under developed econom*” OR “middle income econom*” OR “low income econom*” OR “lower income econom*” OR “low gdp” OR “low gnp” OR “low gross domestic” OR “low gross national” OR “lower gdp” OR “lower gnp” OR “lower gross domestic” OR “lower gross national” OR lmic OR lmics OR “third world” OR “lami countr*” OR “transitional countr*” OR “emerging econom*” OR “emerging nation*”) ) |  |
| S7 | S4 AND S5 AND S6 Limiters - Published Date: 20150101-20231231  Search modes - Boolean/Phrase | 309 |
| **Web of Science Core Collection** | | |
| (((((((((((TS=(“Health auxiliar*” OR “peer group*” OR “health visitor*” OR doula* OR “Allied health personnel” OR “Community health worker*” OR paramedic* OR paraprofessional* “allied health worker*” OR “support worker*” OR “support worker*” OR “home health aide*” OR montrice* OR linkworker* OR “link worker*” OR “barefoot doctor*” OR “expert patient*” OR “health promoter*” OR “health extension worker*” OR “mentor mother*”)) OR TS=((lay OR voluntary OR volunteer* OR untrained OR unlicensed OR "non-professional" OR nonprofessional OR informal OR "non-formal") NEAR/5 (worker* OR visitor* OR attendant* OR aide* OR support* OR person* OR helper* OR carer* OR caregiver* OR "care giver" OR "care givers" OR consultant* OR assistant* OR staff OR visit* OR midwife OR midwives OR provider* OR practitioner*))) OR TS=(trained NEAR/3 (volunteer* OR health NEAR/0 worker* OR mother*))) OR TS=((communit* OR village* OR frontline*) NEAR/3 ((health NEAR/0 worker*) OR (health NEAR/0 care NEAR/0 worker*) OR (healthcare NEAR/0 worker*) OR distributor* OR worker* OR provider*))) OR TS=(communit* NEAR/3 (volunteer* OR aide* OR support))) OR TS=((birth OR childbirth OR labor OR labour) NEAR/1 (attendant* OR assistant*))) OR TS=((lay OR peer) NEAR/1 (volunteer* OR mentor* OR counsel* OR support OR intervention*))) OR TS=((church NEAR/0 based NEAR/3 (intervention* OR program* OR counsel*)))) OR TS=(((health OR healthcare) NEAR/1 outreach))) OR TS=((home NEAR/1 (care OR aide* OR nursing OR support OR intervention* OR treatment* OR visit*)))) OR TS=(((care OR aide* OR nursing OR support OR intervention* OR treatment* OR visit*) NEAR/3 (lay OR volunteer* OR voluntary)))) OR TS=((Auxiliary NEAR/3 (worker* OR nurse* OR midwives OR midwife))) | | |
| AND | | |
| ((((TI=(economic* OR cost OR costs OR costly OR costing OR price OR prices OR pricing OR expenditure OR expenditures OR expense OR expenses OR financial OR finance OR finances OR financed) OR AK=(economic* OR cost OR costs OR costly OR costing OR price OR prices OR pricing OR expenditure OR expenditures OR expense OR expenses OR financial OR finance OR finances OR financed) OR AB=(cost* NEAR/2 (effective* OR utilit* OR benefit* OR minimi* OR analy* OR outcome OR outcomes)) OR AK=(cost* NEAR/2 (effective* OR utilit* OR benefit* OR minimi* OR analy* OR outcome OR outcomes))) OR TS=((value NEAR/2 (money OR monetary)))) OR TS=(“economic model*”)) OR TS=((decision* NEAR/2 (tree* OR analy* OR model*)))) OR TS=(("Disability-Adjusted Life Years" OR DALYS OR "quality-adjusted life years" OR QALYS OR “Return on investment” OR “multiple criteria decision analysis” or MCDA OR “cost-benefit analysis” OR “Economic evaluation*” OR “economic analys*” OR “Financial planning” OR “financial management”)) | | |
| AND | | |
| (Afghanistan OR Burundi OR “Burkina Faso” OR “Central African Republic” OR Congo OR Eritrea OR Ethiopia OR Guinea OR Gambia OR Guinea-Bissau OR Liberia OR Madagascar OR Mali OR Mozambique OR Malawi OR Niger OR “North Korea” OR Rwanda OR Sudan OR “Sierra Leone” OR Somalia OR “South Sudan” OR “Syrian Arab Republic” OR Chad OR Togo OR Uganda OR Yemen OR Zambia OR Angola OR Benin OR Bangladesh OR Bolivia OR Bhutan OR "cote d'ivoir*" OR "cote d' ivoir*" OR “cote divoir*” OR “cote d ivoir*” OR “ivory coast*” OR Cameroon OR Comoros OR “Cabo Verde” OR Djibouti OR Algeria OR Egypt OR Micronesia OR Ghana OR Honduras OR Haiti OR Indonesia OR India OR Iran OR Kenya OR Kyrgyzstan OR Cambodia OR Kiribati OR Laos OR Lebanon OR “Sri Lanka” OR Lesotho OR Morocco OR Myanmar OR Burma OR Mongolia OR Mauritania OR Nigeria OR Nicaragua OR Nepal OR Pakistan OR Philippines OR “Papua New Guinea” OR "West Bank" OR Gaza OR Senegal OR “Solomon Islands” OR “El Salvador” OR "Sao Tome and Principe" OR Eswatini OR Swaziland OR Tajikistan OR Timor-Leste OR Tunisia OR Tanzania OR Ukraine OR Uzbekistan OR Vietnam OR Vanuatu OR Samoa OR Zimbabwe OR Afghan* OR Burundian* OR Burkinabe* OR “Central African*” OR Congolese OR Eritrean* OR Ethiopian* OR Guinean* OR Gambian* OR Guinea-Bissauan* OR Liberian* OR Malagasy* OR Malian* OR Mozambican* OR Malawian* OR Nigerien* OR “North Korean*” OR Rwandan* OR Sudanese OR “Sierra Leonean*” OR Somali* OR Syrian OR Chadian* OR Togolese OR Ugandan* OR Yemeni* OR Zambian* OR Angolan* OR Benin* OR Bangladeshi* OR Bolivian* OR Bhutanese* OR Ivorian* OR Cameroonian* OR Comorian* OR Cape Verdean* OR Djiboutian* OR Algerian* OR Egyptian* OR Micronesian* OR Ghanaian* OR Honduran* OR Haitian* OR Indonesian* OR (Indian* NOT indiana) OR Iranian* OR Kenyan* OR Kyrgyz* OR kirgiz* OR kirghiz* OR Cambodian* OR Kiribatian* OR Laotian* OR Lebanese OR “Sri Lankan*” OR Basotho* OR Moroccan* OR Burmese OR Mongolian* OR Mauritanian* OR Nigerian* OR Nicaraguan* OR Nepalese OR Pakistani* OR Filipino* OR “Papua New Guinean*” OR Palestinian* OR Senegalese OR “Solomon Islander*” OR Salvadoran* OR Salvadorian* OR Sao Tomean* OR Eswatini* OR swazi* OR swati* OR Tajik* OR Timorese OR Tunisian* OR Tanzanian* OR Ukrainian* OR Uzbek* OR Vietnamese OR Vanuatuan* OR Samoan* OR Zimbabwean* OR africa* OR “arab* countr*” OR “middle east*” OR “global south” OR sahara* OR subsahara* OR magreb* OR maghrib* OR “west indies*” OR caribbean* OR “central america*” OR “latin america*” OR “south america*” OR “central asia*” OR “north asia*” OR “northern asia*” OR “southeastern asia*” OR “south eastern asia*” OR “southeast asia*” OR “south east asia*” OR “west asia*” OR “western asia*” OR “east europe*” OR “eastern europe*” OR “developing countr*” OR “developing nation*” OR “developing population*” OR “developing world” OR “less developed countr*” OR “less developed nation*” OR “less developed world” OR “lesser developed countr*” OR “lesser developed nation*” OR “lesser developed world” OR “under developed countr*” OR “under developed nation*” OR “under developed world” OR “underdeveloped countr*” OR “underdeveloped nation*” OR “underdeveloped world” OR “middle income countr*” OR “middle income nation*” OR “middle income population*” OR “low income countr*” OR “low income nation*” OR “low income population*” OR “lower income countr*” OR “lower income nation*” OR “lower income population*” OR “underserved countr*” OR “underserved nation*” OR “underserved population*” OR “under served population*” OR “under served nation*” OR “deprived countr*” OR “deprived population*” OR “high burden countr*” OR “high burden nation*” OR “countdown countr*” OR “countdown nation*” OR “poor countr*” OR “poor nation*” OR “poor population*” OR “poor world” OR “poorer countr*” OR “poorer nation*” OR “poorer population*” OR “poorer world” OR “developing econom*” OR “less developed econom*” OR “underdeveloped econom*” OR “under developed econom*” OR “middle income econom*” OR “low income econom*” OR “lower income econom*” OR “low gdp” OR “low gnp” | | |
| AND | | |
| 2023 or 2022 or 2021 or 2020 or 2019 or 2018 or 2017 or 2016 or 2015 (Publication Years) | | |
| **Scopus** | | |
| ( TITLE-ABS-KEY ( {Health auxiliary} OR {Health auxiliaries} OR {peer group} OR {peer groups} OR {health visitor} OR {health visitors} OR doula* OR {Allied health personnel} OR {Community health worker} OR {Community health workers} OR paramedic* OR paraprofessional* OR {allied health worker} OR {allied health workers} OR {support worker} OR {support workers} OR {home health aide} OR {home health aides} OR montrice* OR linkworker* OR {link worker} OR {link workers} OR {barefoot doctor} OR {barefoot doctors} {expert patient} OR {expert patients} OR {health promoter} OR {health promoters} OR {health extension worker} OR {health extension workers} OR {mentor mother} OR {mentor mothers} ) OR TITLE-ABS-KEY ( ( lay OR voluntary OR volunteer* OR untrained OR unlicensed OR "non professional" OR nonprofessional* OR informal OR "non-formal" ) W/5 ( worker* OR visitor* OR attendant* OR aide* OR support* OR person* OR helper* OR carer* OR caregiver* OR "care giver" OR "care givers" OR consultant* OR assistant* OR staff OR visit* OR midwife OR midwives OR provider* OR practitioner* ) ) OR TITLE-ABS-KEY ( trained W/3 ( volunteer* OR "health worker" OR "health workers" OR mother* ) ) OR TITLE-ABS-KEY ( ( communit* OR village* OR frontline* ) W/3 ( "health worker" OR "health workers" OR "health care worker" OR "health care workers" OR "healthcare worker" OR "healthcare workers" OR distributor* OR worker* OR provider* ) ) OR TITLE-ABS-KEY ( communit* W/3 ( volunteer* OR aide* OR support ) ) OR TITLE-ABS-KEY ( ( birth OR childbirth OR labor OR labour ) W/1 ( attendant* OR assistant* ) ) OR TITLE-ABS-KEY ( ( lay OR peer ) W/1 ( volunteer* OR mentor* OR counsel* OR support OR intervention* ) ) OR TITLE-ABS-KEY ( "church based" W/3 ( intervention* OR program* OR counsel* ) ) OR TITLE-ABS-KEY ( ( health OR healthcare ) W/1 outreach ) OR TITLE-ABS-KEY ( home W/1 ( care OR aide* OR nursing OR support OR intervention* OR treatment* OR visit* ) ) OR TITLE-ABS-KEY ( ( care OR aide* OR nursing OR support OR intervention* OR treatment* OR visit* ) W/3 ( lay OR volunteer* OR voluntary ) ) OR TITLE-ABS-KEY ( auxiliary W/3 ( worker* OR nurse* OR midwives OR midwife ) ) ) AND ( TITLE ( economic* OR cost OR costs OR costly OR costing OR price OR prices OR pricing OR expenditure OR expenditures OR expense OR expenses OR financial OR finance OR finances OR financed ) OR KEY ( economic* OR cost OR costs OR costly OR costing OR price OR prices OR pricing OR expenditure OR expenditures OR expense OR expenses OR financial OR finance OR finances OR financed ) OR ABS ( cost* W/2 ( effective* OR utilit* OR benefit* OR minimi* OR analy* OR outcome OR outcomes ) ) OR KEY ( cost* W/2 ( effective* OR utilit* OR benefit* OR minimi* OR analy* OR outcome OR outcomes ) ) OR TITLE-ABS-KEY ( value W/2 ( money OR monetary ) ) OR TITLE-ABS-KEY ( "economic model" OR "economic models" ) OR TITLE-ABS-KEY ( decision* W/2 ( tree* OR analy* OR model* ) ) OR TITLE-ABS-KEY ( "Disability-Adjusted Life Years" OR dalys OR "quality-adjusted life years" OR qalys OR "Return on investment" OR "multiple criteria decision analysis" OR mcda OR "cost-benefit analysis" OR "Economic evaluation" OR "Economic evaluations" OR "economic analysis" OR "economic analyses" OR "Financial planning" OR "financial management" ) ) AND ( TITLE-ABS-KEY ( ( afghanistan OR burundi OR "Burkina Faso" OR "Central African Republic" OR congo OR eritrea OR ethiopia OR guinea OR gambia OR guinea-bissau OR liberia OR madagascar OR mali OR mozambique OR malawi OR niger OR "North Korea" OR rwanda OR sudan OR "Sierra Leone" OR somalia OR "South Sudan" OR "Syrian Arab Republic" OR chad OR togo OR uganda OR yemen OR zambia OR angola OR benin OR bangladesh OR bolivia OR bhutan OR "cote d'ivoir*" OR "cote d' ivoir*" OR "cote divoir*" OR "cote d ivoir*" OR "ivory coast*" OR cameroon OR comoros OR "Cabo Verde" OR djibouti OR algeria OR egypt OR micronesia OR ghana OR honduras OR haiti OR indonesia OR india OR iran OR kenya OR kyrgyzstan OR cambodia OR kiribati OR laos OR lebanon OR "Sri Lanka" OR lesotho OR morocco OR myanmar OR burma OR mongolia OR mauritania OR nigeria OR nicaragua OR nepal OR pakistan OR philippines OR "Papua New Guinea" OR "West Bank" OR gaza OR senegal OR "Solomon Islands" OR "El Salvador" OR "Sao Tome and Principe" OR eswatini OR swaziland OR tajikistan OR timor-leste OR tunisia OR tanzania OR ukraine OR uzbekistan OR vietnam OR vanuatu OR samoa OR zimbabwe OR afghan* OR burundian* OR burkinabe* OR "Central African*" OR congolese OR eritrean* OR ethiopian* OR guinean* OR gambian* OR guinea-bissauan* OR liberian* OR malagasy* OR malian* OR mozambican* OR malawian* OR nigerien* OR "North Korean*" OR rwandan* OR sudanese OR "Sierra Leonean*" OR somali* OR syrian OR chadian* OR togolese OR ugandan* OR yemeni* OR zambian* OR angolan* OR benin* OR bangladeshi* OR bolivian* OR bhutanese* OR ivorian* OR cameroonian* OR comorian* OR cape AND verdean* OR djiboutian* OR algerian* OR egyptian* OR micronesian* OR ghanaian* OR honduran* OR haitian* OR indonesian* OR ( indian* AND not AND indiana ) OR iranian* OR kenyan* OR kyrgyz* OR kirgiz* OR kirghiz* OR cambodian* OR kiribatian* OR laotian* OR lebanese OR "Sri Lankan*" OR basotho* OR moroccan* OR burmese OR mongolian* OR mauritanian* OR nigerian* OR nicaraguan* OR nepalese OR pakistani* OR filipino* OR "Papua New Guinean*" OR palestinian* OR senegalese OR "Solomon Islander*" OR salvadoran* OR salvadorian* OR sao AND tomean* OR eswatini* OR swazi* OR swati* OR tajik* OR timorese OR tunisian* OR tanzanian* OR ukrainian* OR uzbek* OR vietnamese OR vanuatuan* OR samoan* OR zimbabwean* OR africa* OR "arab* countr*" OR "middle east*" OR "global south" OR sahara* OR subsahara* OR magreb* OR maghrib* OR "west indies*" OR caribbean* OR "central america*" OR "latin america*" OR "south america*" OR "central asia*" OR "north asia*" OR "northern asia*" OR "southeastern asia*" OR "south eastern asia*" OR "southeast asia*" OR "south east asia*" OR "west asia*" OR "western asia*" OR "east europe*" OR "eastern europe*" OR "developing countr*" OR "developing nation*" OR "developing population*" OR "developing world" OR "less developed countr*" OR "less developed nation*" OR "less developed world" OR "lesser developed countr*" OR "lesser developed nation*" OR "lesser developed world" OR "under developed countr*" OR "under developed nation*" OR "under developed world" OR "underdeveloped countr*" OR "underdeveloped nation*" OR "underdeveloped world" OR "middle income countr*" OR "middle income nation*" OR "middle income population*" OR "low income countr*" OR "low income nation*" OR "low income population*" OR "lower income countr*" OR "lower income nation*" OR "lower income population*" OR "underserved countr*" OR "underserved nation*" OR "underserved population*" OR "under served population*" OR "under served nation*" OR "deprived countr*" OR "deprived population*" OR "high burden countr*" OR "high burden nation*" OR "countdown countr*" OR "countdown nation*" OR "poor countr*" OR "poor nation*" OR "poor population*" OR "poor world" OR "poorer countr*" OR "poorer nation*" OR "poorer population*" OR "poorer world" OR "developing econom*" OR "less developed econom*" OR "underdeveloped econom*" OR "under developed econom*" OR "middle income econom*" OR "low income econom*" OR "lower income econom*" OR "low gdp" OR "low gnp" OR "low gross domestic" OR "low gross national" OR "lower gdp" OR "lower gnp" OR "lower gross domestic" OR "lower gross national" OR lmic OR lmics OR "third world" OR "lami countr*" OR "transitional countr*" OR "emerging econom*" OR "emerging nation*" ) ) ) AND PUBYEAR > 2014 AND PUBYEAR < 2024 | | |
| **LILACS** | | |
| tw:'Health auxiliary' OR tw:'Health auxiliaries' OR tw:'peer group' OR tw:'peer groups' OR tw:'health visitor' OR tw:'health visitors' OR tw:doula* OR tw:'Allied health personnel' OR tw:'Community health worker' OR tw:'Community health workers' OR tw:paramedic* OR tw:paraprofessional* OR tw:'allied health worker' OR tw:'allied health workers' OR tw:'support worker' OR 'tw:support workers' OR tw:'home health aide' OR tw:'home health aides' OR tw:montrice* OR tw:linkworker* OR tw:'link worker' OR tw:'barefoot doctor' OR tw:'barefoot doctors' OR tw:'expert patient' OR tw:'expert patients' OR tw:health promoter* OR tw:'health extension worker' OR tw:'health extension workers' OR tw:'mentor mother' OR tw:'mentor mothers'OR tw:carer* OR tw:caregiver* OR tw:'care giver' OR tw:midwife OR tw:midwives OR tw:paraprofessional* OR tw:paramedic* OR tw:'allied health worker' OR tw:'allied health workers' OR tw:'support worker' OR tw:'support workers' OR tw:'trained volunteer' OR tw:'trained volunteers' OR tw:'trained health worker' OR tw:'trained health workers' OR tw:'trained mother' OR tw:'trained mothers' OR tw:'community health care worker' OR tw:'community health care workers' OR tw:'community healthcare worker' OR tw:'community healthcare workers' OR tw:'village health worker' OR tw:'village health workers' OR tw:'frontline provider' OR tw:'frontline providers' OR tw:'community volunteer' OR tw:'community volunteers' OR tw:'community aide' OR tw:'community aides' OR tw:'community support' OR tw:'birth attendant' OR tw:'birth attendants' OR tw:'birth assistant' OR tw:'birth assistants' OR tw:'childbirth attendant' OR tw:'childbirth attendants' OR tw:'childbirth assistant' OR tw:'childbirth assistants' OR tw:'labor attendant' OR tw:'labor attendants' OR tw:'labor assistant' OR tw:'labor assistants' OR tw:'labour attendant' OR tw:'labour attendants' OR tw:'labour assistant' OR tw:'labour assistants' OR tw:'lay volunteer' OR tw:'lay volunteers' OR tw:'lay counselor' OR tw:'lay counselors' OR tw:'peer volunteer' OR tw:'peer volunteers' OR tw:'peer support' OR tw:'peer mentor' OR tw:'peer mentors' OR tw:'church based intervention' OR tw:'church based interventions' OR tw:'church based program' OR tw:'church based programs' OR tw:'church based counseling' OR tw:'health outreach' OR tw:'healthcare outreach' OR tw:'home care' OR tw:'home aide' OR tw:'home visit' OR tw:'home visits' OR tw:'volunteer nurse' OR tw:'volunteer nurses' OR tw:'auxiliary worker' OR tw:'auxiliary workers' OR tw:'auxiliary nurse' OR tw:'auxiliary nurses' OR tw:'health promoter' OR tw:'health promoters' | | |
