# Supplementary material for: Costs and cost-effectiveness of community health worker programs focussed on HIV, TB and malaria infectious diseases in low- and middle-income countries (2015–2024): A scoping literature review
Source: PLOS Glob Public Health. 2025 May 9;5(5):e0004596. doi: 10.1371/journal.pgph.0004596 (PMC12063845; doi:10.1371/journal.pgph.0004596)
Supplement: Attachment — Submitted filename: Review PGPH-D-25-00054.docx [file pgph.0004596.s005.docx]

Review for PGPH-D-25-00054

I appreciate the opportunity to review this manuscript. Below, I provide my evaluation based on the significance of the claims, contextualization within the literature, and robustness of the data and analyses. I also offer minor editorial suggestions.

Significance of the claims

The manuscript’s central claim is that CHW programs are generally cost-effective compared to facility-based care, particularly in improving treatment adherence and targeting high-priority populations. It also highlights methodological heterogeneity across studies, which limits direct comparisons. Given the increasing recognition of CHWs as essential to primary healthcare in LMICs, this review is highly relevant for policymakers and donors considering CHW investment.

Placement within the existing literature

The manuscript builds effectively on prior research, particularly Vaughan et al., by incorporating newer evidence and addressing methodological gaps. The literature review is thorough, drawing from both peer-reviewed and grey literature. However, additional contextualization would further strengthen the discussion:

- **Comment 1:** Engagement with CHW Investment Cases
  - The manuscript does not sufficiently engage with existing investment cases for CHWs, many of which include cost-effectiveness analyses. Discussing how these findings align with or differ from such investment cases would add valuable context.
  - Several CHW investment cases in LMICs include cost-effectiveness and budget impact analysis results. Have these been considered? While some provide horizontal evidence, others may offer disaggregated results.
  - *Page 46:* The statement—"Given the growing evidence that CHW programs are cost-effective, future research should prioritize budget impact analyses or other methods specifically tailored to assessing affordability, rather than focusing solely on costs and cost-effectiveness"—should acknowledge the substantial number of CHW investment cases already available.
- **Comment 2:** Recent Africa CDC review
  - The *Africa CDC Health Economics Division* recently conducted a review on the cost-effectiveness of CHW program components. While I have not seen the results, it may be valuable to explore potential synergies between this review and your analysis.
  - Expanding on how research gaps (e.g., affordability and integrated CHW models) could be addressed—such as through collaborations with *Africa CDC* or *Ministries of Health*—would enhance the paper’s impact.

Do the data and analyses support the claims?

The study follows a transparent methodology for study selection and data extraction. The categorization of cost outcomes is particularly useful for policy interpretation. However, several limitations should be addressed:

- **Comment 3:** Justification for relying on authors’ cost-effectiveness and affordability conclusions (*page 13*)
  - The manuscript clarifies that it reports only the original authors’ assessments of cost-effectiveness and affordability, noting this as a limitation. However, it would be helpful to briefly justify this decision. Could a reassessment be possible using publicly available data? If not, is this an area for future research? The absence of *CHEERS checklists* in many studies is mentioned—does this make independent assessment infeasible?
- **Comment 4:** CHW compensation data
  - The manuscript frequently notes that CHW remuneration details are unavailable. However, in some cases, could this information be obtained from publicly available sources such as national health budgets? If this approach was considered and deemed unfeasible, clarifying why would strengthen the discussion.

Minor Edits

- **Comment 5: Page 5:** The term *"disease mortality rates"* is used, but the cited data refers to the absolute *number of deaths.*
- **Comment 6: Page 5:** Consider adding the term *"inequities"* alongside *"health inequalities"*, as inequities imply avoidable and unjust differences.
- **Comment 7: Page 12:** In the section *"(iii) Mortality and Morbidity Outcomes,"* include an example of a morbidity outcome.
- **Comment 8: Page 13:** Typo: *"ee"* should be *"we."*
- **Comment 9: Page 28:** The manuscript states, *"A study conducted in Tanzania found HIV testing and counseling delivered by CHWs to be most cost-effective when delivered in a facility vs. at home or in a public venue."* Consider clarifying in the inclusion/exclusion criteria that CHWs can be based in facilities as well as in the community, as this is a common misconception.
- **Comment 10: Page 44:** The manuscript notes that since the 2015 review, the number of economic studies on vertical CHW programs for HIV, malaria, and TB has significantly increased. It would be valuable to comment on potential reasons—e.g., an increase in CHW-delivered services or a general rise in cost-effectiveness studies?
- **Comment 11: Page 45-46:** The transition between the last sentence on *page 45* and the first sentence on *page 46* could be clarified for readability.
- **Comment 12: General:** There is a significant shortfall of CHWs in LMICs. While there is evidence that CHWs are cost-effective, generate a strong return on investment, and are affordable, they remain underutilized. It may be useful to highlight this in the introduction or discussion.
- **Comment 13: Throughout:** The paper discusses disputes over cost-effectiveness thresholds. Consider citing: Pichon-Riviere et al. DOI: [10.1016/S2214-109X(23)00162-6](https://doi.org/10.1016/s2214-109x(23)00162-6) and Ochalek et al. DOI: [10.1136/bmjgh-2018-000964](https://doi.org/10.1136/bmjgh-2018-000964)

PLOS guidance for reviews:

- [The study presents the results of original research.](https://journals.plos.org/globalpublichealth/s/criteria-for-publication#loc-1)
  - Yes – to my knowledge.
- [Results reported have not been published elsewhere](https://journals.plos.org/globalpublichealth/s/criteria-for-publication#loc-2).
  - Yes – to my knowledge
- [Experiments, statistics, and other analyses are performed to a high technical standard and are described in sufficient detail.](https://journals.plos.org/globalpublichealth/s/criteria-for-publication#loc-3)
  - Yes
- [Conclusions are presented in an appropriate fashion and are supported by the data](https://journals.plos.org/globalpublichealth/s/criteria-for-publication#loc-4).
  - Yes, but clarification for point listed in my comments
- [The article is presented in an intelligible fashion and is written in standard English](https://journals.plos.org/globalpublichealth/s/criteria-for-publication#loc-5).
  - Yes
- [The research meets all applicable standards for the ethics of experimentation and research integrity](https://journals.plos.org/globalpublichealth/s/criteria-for-publication#loc-6).
  - Yes
- [The article adheres to appropriate reporting guidelines and community standards for data availability](https://journals.plos.org/globalpublichealth/s/criteria-for-publication#loc-7).
  - Yes
- What are the main claims of the paper and how significant are they for the discipline?
  - Addressed above.
- Are the claims properly placed in the context of the previous literature? Have the authors treated the literature fairly?
  - Yes, suggested changes addressed above.
- Do the data and analyses fully support the claims? If not, what other evidence is required?
  - Yes, suggested changed addressed above.
- If a protocol is provided, for example for a randomized controlled trial, are there any important deviations from it? If so, have the authors explained adequately why the deviations occurred?
  - NA
- PLOS Global Public Health encourages authors to publish detailed protocols and algorithms as supporting information online. Do any particular methods used in the manuscript warrant such treatment?
  - They have provided this
- If the paper is considered unsuitable for publication in its present form, does the study itself show sufficient potential that the authors should be encouraged to resubmit a revised version?
  - NA
- Are original data deposited in appropriate repositories and accession/version numbers provided for genes, proteins, mutants, diseases, etc.?
  - NA
- Does the study conform to any [relevant guidelines](https://journals.plos.org/globalpublichealth/s/best-practices-in-research-reporting#loc-reporting-guidelines-for-specific-research-resources-and-study-types) such as CONSORT, MIAME, QUORUM, STROBE, and the [Fort Lauderdale agreement](https://www.sanger.ac.uk/wp-content/uploads/fortlauderdalereport.pdf)?
  - Yes - PRISMA
- Are details of the methodology sufficient to allow the experiments to be reproduced?
  - Yes
- Is any software created by the authors freely available?
  - No - NA

1. Is the manuscript well organized and written clearly enough to be accessible to non-specialists?
   a. Yes
