# Supplementary material for: Costs and cost-effectiveness of community health worker programs focussed on HIV, TB and malaria infectious diseases in low- and middle-income countries (2015–2024): A scoping literature review
Source: PLOS Glob Public Health. 2025 May 9;5(5):e0004596. doi: 10.1371/journal.pgph.0004596 (PMC12063845; doi:10.1371/journal.pgph.0004596)
Supplement: Attachment — Submitted filename: Infectious disease paper_ Response to Reviewers-2.docx [file pgph.0004596.s006.docx]

**Response to Reviewer Comments**

Dear Reviewer,

We sincerely appreciate your thoughtful feedback and constructive suggestions to improve the clarity and rigor of our manuscript. Below, we provide our responses to each of your comments and outline the revisions we have made accordingly.

### **Response to Comment 1: Engagement with CHW Investment Cases**

Thank you for this important suggestion. While several CHW investment cases provide valuable insights into cost-effectiveness and ROI, they did not meet our inclusion criteria, which required explicit empirical economic evaluations of CHW programs for HIV, malaria, or TB in LMICs published between 2015 and 2024.

However, we recognize the significance of these reports, particularly the 2013 One Million CHWs Report, the 2015 CHW Financing Framework, and ongoing unpublished global ROI analyses. While these studies have shaped the global discourse on CHW investments, they often lack methodological transparency, comparability across settings, and empirical validation at the country level—key concerns raised by policymakers. In our experience, MoHs often question the applicability of broad ROI figures (e.g., the 10:1 return estimate) to their specific contexts and require more rigorous, locally grounded evidence before making financing decisions.

Our study complements these investment cases by systematically analyzing empirical economic evaluations and identifying research gaps, such as the lack of CHW remuneration data, affordability assessments, and standardized cost-effectiveness reporting. By strengthening methodological completeness and transparency, our findings serve as a foundation for future country-specific modeling efforts.

To reflect this, we have expanded the Discussion and Directions for Future Research sections to:

1. Acknowledge the contributions and limitations of existing investment cases while emphasizing the need for more rigorous, country-specific analyses.
2. Position our review as a methodological backbone for policymakers and funders seeking empirical validation of CHW cost-effectiveness beyond broad global ROI estimates.
3. Highlight the importance of integrating real-world financing data (e.g., national health budgets, donor contributions, and cost-sharing models) to bridge the gap between research and policy planning.

### **Response to Comment 2: Recent Africa CDC Review**

We are grateful to the reviewer for highlighting the ongoing review conducted by the Africa CDC Health Economics Division on the cost-effectiveness of CHW program components. While the full results of this review were not publicly available at the time of our study, we acknowledge its potential to complement our findings and inform national financing decisions.

To address this, we have:

1. Incorporated a discussion of Africa CDC’s work in the Directions for Future Research section and flagged potential synergies.
2. Suggested pathways for collaboration with regional health bodies, including Africa CDC and Ministries of Health, to translate country-specific findings into actionable policy recommendations.

Additionally, we have highlighted that our supplementary materials provide disaggregated country-level data, which can be leveraged by policy organizations such as the Community Health Impact Coalition (CHIC) to support Ministries of Health in designing and financing CHW programs.

### **Response to Comment 3: Justification for Relying on Authors’ Cost-Effectiveness and Affordability Conclusions**

Thank you for this thoughtful suggestion. We chose to rely on the original authors’ assessments to maintain objectivity, as our study follows a scoping review approach, which is designed to map existing evidence rather than critically reassess methodologies.

A reassessment using publicly available data was not feasible due to variability in study methodologies and the absence of a standardized reporting framework for CHW economic evaluations. While tools like CHEERS exist, they are not consistently applied, making independent evaluation challenging.

We have emphasized in the Directions for Future Research section the importance of improving reporting transparency and developing standardized approaches to strengthen the comparability and utility of cost-effectiveness and affordability assessments.

### **Response to Comment 4: CHW Compensation Data**

Thank you for raising this important point. Extracting and standardizing this information across multiple countries was beyond the scope of our scoping review, which aimed to synthesize findings from published economic evaluations rather than conduct independent cost analyses. Additionally, CHIC’s prior transparency assessment of major global health funders found that detailed CHW compensation data is largely absent from public reporting. We have now clarified in the Discussion that future research should integrate national financing data where available and advocate for improved reporting on CHW remuneration to bridge the gap between cost-effectiveness studies and real-world policy planning.

### **Minor Edits**

- **Comment 5 (Page 5: “Disease mortality rates” vs. absolute number of deaths)**
  Thank you for catching this inconsistency. We have revised the text to clarify that the cited data refer to the absolute number of deaths rather than mortality rates.
- **Comment 6 (Page 5: Adding “inequities” alongside “health inequalities”)**
  We appreciate this suggestion and have incorporated the term “inequities” alongside “health inequalities” to reflect avoidable and unjust differences in health outcomes.
- **Comment 7 (Page 12: Adding an example of a morbidity outcome)**
  We have added a specific morbidity outcome example to clarify this section.
- **Comment 8 (Page 13: Typo “ee” should be “we”)**
  Thank you for pointing this out. We have corrected this typographical error.
- **Comment 9 (Page 28: Clarifying that CHWs can be based in facilities as well as in the community)**
  Thank you for this suggestion. Rather than updating the inclusion/exclusion criteria retrospectively, we have instead clarified this point by modifying the definition of CHWs to explicitly state that they may operate in both facility and community settings. We believe this revision ensures that readers can accurately interpret the scope of CHW roles without requiring changes to the study criteria.
- **Comment 10 (Page 44: Providing potential reasons for the increase in CHW economic studies since 2015)**
  This is an excellent point. We have expanded the discussion to note that the increase in economic evaluations of CHW programs may be attributed to the growing role of CHWs in differentiated service delivery and the broader emphasis on cost-effectiveness research in global health.
- **Comment 11 (Pages 45-46: Clarifying the transition between sentences for readability)**
  We have revised the transition to improve clarity and ensure a smoother flow between the last sentence on page 45 and the first sentence on page 46.
- **Comment 12 (General: Highlighting the underutilization of CHWs despite cost-effectiveness evidence)**
  We agree that this is a critical point. We have incorporated a sentence into the Future Research section to explicitly address the issue of CHW workforce shortages and their limited integration into national health systems, despite strong evidence of their cost-effectiveness.
- **Comment 13 (Throughout: Citing relevant literature on cost-effectiveness thresholds)**
  Thank you for these recommendations. We have now cited Pichon-Riviere et al. (2023) and Ochalek et al. (2018) in relevant sections discussing cost-effectiveness thresholds and their limitations.

**Reviewer 2**

Comment - Page 13: there appears to be a typing error at the end of the second paragraph "That said, ee report"

Response: This has been identified by R1 and revised accordingly.

Figure 1: some of the numbers don't seem to add up as I expected, e.g. 5663 records screened minus 5345 records excluded gives 318 rather than the 316, then minus the additional 170 excluded gives a number higher than the final 33 included.

Response: Thank you for identifying this error. We have now updated and revised Figure 1 to reflect the true numbers.

We greatly appreciate your insightful comments, which have helped to strengthen the manuscript. Please let us know if any further clarifications or revisions are needed.

Best regards,
James O’Donovan (on behalf of all authors)
